# Associations of prognostic nutritional index with cardiovascular all-cause mortality among CVD patients with diabetes or prediabetes: evidence from the NHANES 2005–2018

**DOI:** 10.3389/fimmu.2025.1518295

**Published:** 2025-02-12

**Authors:** WenYi Xu, Li Zhang, QianKun Yang, Ying Cao, Rui Rao, Li Lv, Qin Cen, Qiong Wei, LuLing Yang

**Affiliations:** ^1^ Department of Pediatrics, The People’s Hospital of Leshan, Leshan, Sichuan, China; ^2^ Ministry of Education Key Laboratory of Child Development and Disorders, Department of Neurosurgery, Children’s Hospital of Chongqing Medical University, National Clinical Research Center for Child Health and Disorders, Chongqing, China; ^3^ Chongqing Key Laboratory of Pediatrics, Department of Neurosurgery, Children’s Hospital of Chongqing Medical University, National Clinical Research Center for Child Health and Disorders, Chongqing, China; ^4^ National & Regional United Engineering Lab of Tissue Engineering, Department of Orthopedics, Southwest Hospital, Third Military Medical University (Army Medical University), Chongqing, China

**Keywords:** prognostic nutritional index, cardiovascular disease, diabetes, prediabetes, mortality

## Abstract

**Background:**

Immunonutritional status is linked to the prognosis of cardiovascular disease (CVD) and diabetes, but the relationship between immunonutritional disorders and clinical outcomes in CVD patients with diabetes is unclear. This study aims to investigate the association of the novel immunonutritional indicator of prognostic nutritional index (PNI) with all-cause and CVD mortality in diabetic and prediabetic CVD patients.

**Method:**

This is an open-cohort study involving 1,509 CVD patients with diabetes or prediabetes collected from The National Health and Nutrition Examination Survey (NHANES) and initially interviewed between 2005 and 2018. Subjects were followed up until on December 31, 2019. Mortality outcomes and causes of death were obtained from National Death Index (NDI) records. We used restricted cubic spline (RCS) and maximally selected rank statistics method (MSRSM) to assess the nonlinearity of the PNI-mortality association and determine the optimal PNI cutoff for survival outcomes. Additionally, weighted multivariable Cox regression models, subgroup analyses, and interaction tests were employed to examine the relationship between PNI and all-cause and CVD mortality. The predictive accuracy of PNI for survival outcomes was evaluated using time-dependent receiver operating characteristic curve (ROC) analysis.

**Results:**

During a median follow-up of 61 months (interquartile range, 33-103 months), 507 of the 1509 (33.60%) diabetic or prediabetic CVD patients died. A negative and nonlinear association between PNI and all-cause/CVD mortality was identified by RCS analysis in all patients. In the fully-adjusted Cox regression model, in the entire cohort, higher PNI (≥46.5) was significantly associated with reduced risks for all-cause and CVD mortality. A consistent association between PNI and all-cause/CVD mortality was observed in diabetic CVD patients, but not in prediabetic CVD patients. No significant interaction between PNI and other covariates was observed (all P interaction >0.05). Time-dependent ROC curve revealed that the areas under the curve (AUC) of PNI for 1-, 3-, 5-, and 10-year survival rates were 0.66, 0.66, 0.66, and 0.67 for all-cause mortality, and 0.72, 0.70, 0.72, and 0.69 for CVD mortality, respectively.

**Conclusion:**

Increased PNI is significantly associated with reduced risks for all-cause and CVD mortality in diabetic or prediabetic CVD patients, especially for diabetic CVD patients.

## Introduction

According to the Global Burden of Diseases, Injuries, and Risk Factors Study, which has monitored trends in mortality and disability since 1990, cardiovascular disease (CVD) continues to be the primary cause of illness and death globally (1). In 2019, the global prevalence of cardiovascular disease (CVD) across 204 countries and regions rose dramatically from 271 million in 1990 to 523 million. Similarly, the number of CVD-related deaths increased from 12.1 million to 18.6 million during the same period, solidifying CVD as a leading cause of mortality worldwide (2). Abundant evidences have revealed that type 2 diabetes mellitus (T2DM) and pre-diabetes are usually occurred as one of the comorbidities in CVD patients, and usually correlated with poor outcomes (3). Therefore, exploring the prognostic factors for CVD patients with different glucose metabolism statuses is crucial for assessing the risk of mortality, especially the risk of CVD mortality.

Numerous studies have shown that diabetes and dysglycemia significantly increase the risk of cardiovascular events due to lipid metabolism abnormalities. In patients with CVD and diabetes, the risk of myocardial infarction and sudden cardiac death is 2 to 3 times higher than in those with CVD alone. Therefore, exploration of the prognosticators related to individuals with CVD and diabetes is essential for improving prognosis. Malnutrition is a prevalent and critical concern among patients with type 2 diabetes mellitus, particularly those with cardiovascular disease (CVD). While the precise prevalence of malnutrition in this population remains ambiguous, studies indicate that over 50% are either malnourished or at risk (4–6), with elderly individuals being especially susceptible. For example, a survey involving 1,090 hospitalized older diabetic patients in Spain revealed that 39.1% were at risk of malnutrition, while 21.2% were already malnourished (5). Recent studies have suggested that nutritional deficiencies in diabetic patients are associated with declines in activities of daily living, grip strength, physical performance, and overall quality of life, as well as prolonged hospital stays and elevated mortality rates (4, 7, 8). These findings suggest that malnutrition may exacerbate the progression of cardiovascular disease and contribute to unfavorable health outcomes. Consequently, early identification and intervention for malnutrition are imperative to enhance the prognosis for patients with diabetes and CVD.

The Prognostic Nutritional Index (PNI), which is calculated based on serum albumin and lymphocyte counts, serves as an immunonutritional marker that reflects the body’s chronic inflammation, immune function, and nutritional status (9). Numerous studies have demonstrated the utility of PNI as a prognostic indicator in various diseases, including malignancies such as gastrointestinal cancer (10), lymphoma (11), and oral cancer (12), as well as benign conditions like hip fractures (13), Crohn’s disease (14), and ischemic stroke (15). Recent research has shown that a higher PNI is associated with a lower risk of diabetic kidney disease (OR=0.64, 95% CI: 0.459-0.892, P=0.01) and reduced all-cause mortality (HR=0.60, 95% CI: 0.37-0.97, P=0.036) in patients with type 2 diabetes mellitus (T2DM) (16). Other studies have also indicated that lower PNI levels significantly increase the risks of all-cause and cardiovascular disease (CVD) mortality in T2DM and gestational diabetes patients (17, 18). Additionally, PNI has been reported to provide superior predictive value for all-cause and CVD mortality in the general population compared to other nutritional biomarkers, such as the geriatric nutritional risk index, controlling nutritional status score, triglycerides, total cholesterol, and body weight index (19). These findings underscore the significant prognostic value of PNI for patients with diabetes or CVD. However, it remains unclear whether PNI as an immunonutritional biomarker is associated with the prognosis of CVD patients who also have diabetes or prediabetes.

Therefore, the aim of the current study was to explore the relationship of PNI with all-cause and CVD mortality risk in a large, nationally representative sample of diabetic and prediabetic CVD patients.

## Methods

### Study design

This open-cohort study involved 1,509 participants with diabetes or prediabetes. Data were collected from the National Health and Nutrition Examination Survey (NHANES) for individuals initially interviewed between 2005 and 2018 and were subsequently linked to the National Death Index (NDI) of the National Center for Health Statistics (NCHS) to assess survival status, with follow-up until December 31, 2019. NHANES, managed by the Centers for Disease Control and Prevention (CDC), is a cross-sectional initiative designed to evaluate the health status of a representative U.S. population sample using weighted survey data from interviews, examinations, dietary assessments, and laboratory tests. Detailed information about NHANES can be found in other studies (20). The initial survey protocol received approval from the NCHS Institutional Review Board, and all participants provided informed consent (20).

### Study population

**Figure 1:**
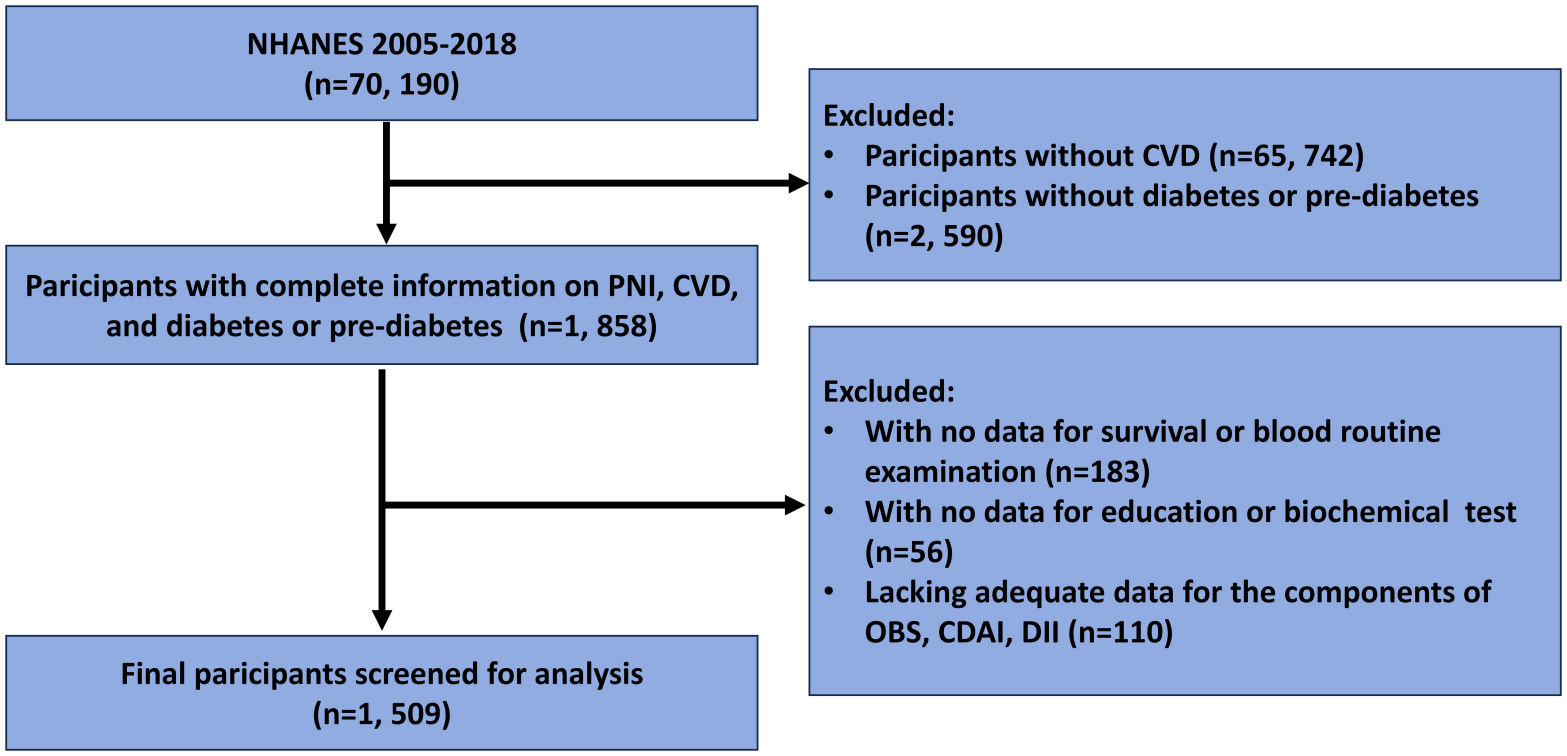
Flow chart for participants selection.

In this study, participants who were surveyed between 2005 and 2018 and identified as having both CVD and diabetes/pre-diabetes (including all existing patients) were included. Those with incomplete information on PNI, CVD, diabetes or prediabetes, all-cause mortality, and other essential data (including blood routine test, education, biochemical test, etc.) were excluded. Finally, a total of 1, 509 participants were included in the current study (**Figure 1**). According to the diabetes diagnostic criteria from American Diabetes Association (ADA), diabetes can be identified by the following criteria, including self-reported diagnosis, use of insulin or oral hypoglycemic medication, plasma fasting glucose levels ≥126 mg/dL (7.0 mmol/L), random blood glucose or 2h oral glucose tolerance test blood glucose≥200 mg/dL (11.1 mmol/L), or HbA1c level ≥6.5% (21, 22). Similarly, prediabetes can be identified by the following criteria, including self-reported prediabetes status or having FBG between 100 mg/dL and 125 mg/dL, or HbA1c between 5.7% and 6.4% (21–23). The identification of CVD was determined through self-reported diagnoses from physicians, which were collected during personal interviews utilizing a standardized questionnaire focused on medical conditions. Participants were asked by the question of “Has a doctor or other health expert ever informed you that you have CHF/CHD/angina pectoris/myocardial infarction (MI)/stroke?”, and those who answered “yes” to any of the above questions were classified as having CVD.

### Assessment of PNI

**Figure 2:**
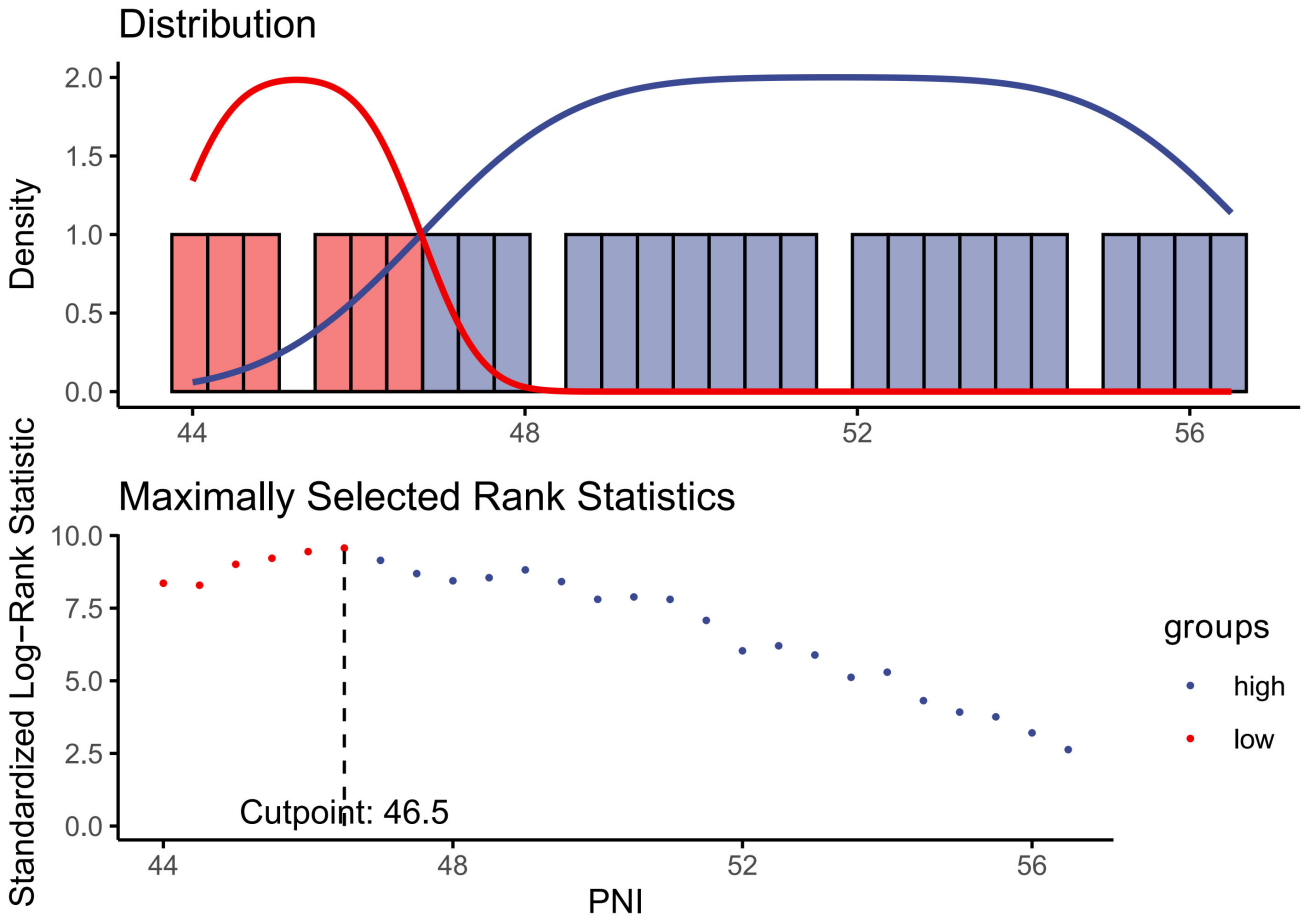
Identification of the cut-off value of PNI based on the method of maximally selected rank statistics.

PNI is calculated using the formula provided below, which is based on the relevant information obtained during the initial interviews of individuals (24): PNI =  serum albumin (g/L) + 5 × total lymphocyte counts (10^9^/L). In order to explore the association between PNI and CVD/all-cause mortality, The participants were classified into three groups and two group based on the PNI’s tertile (lower tertile, middle tertile, upper tertile) and the cut-off (<46.5, ≥46.5) value calculated by using the maximally selected log-rank statistics based on the ‘maxstat’ package (**Figure 2**), respectively. The PNI lower tertile group and PNI<46.5 group was utilized as the reference group.

### Assessment of covariates

Based on previous studies, some pivotal and essential covariates were collected in this study, including demographic data, behavioral risk factors, and chronic non-communicable diseases, and laboratory test data (25–27). The demographic data including age (<60, ≥60), gender (male, female), race (non-Hispanic White, non-Hispanic Black, Hispanic, others), education (less than high school, high school or equivalent, college or above), family poverty income ratio (≤1.0, 1.0-3.0, ≥3.0), and BMI (<25, 25-30, ≥30). The cut-off values for age and PIR in subgroup definition were based on previous studies (26, 27), while the cut-off value for BMI was based on the criterion from World Health Organization (WHO) (28). Additional covariates included participants’ behavioral risk factors such as such as smoking (21) and drinking (27), and the disease status including hypertension and chronic obstructive pulmonary disease (COPD). Smoking status, as described in previous studies (21), was classified into three categories: never smoker (defined as having smoked fewer than 100 cigarettes in a lifetime), former smoker (having smoked more than 100 cigarettes but currently not smoking), and current smoker (having smoked more than 100 cigarettes and currently smoking either occasionally or daily). Alcohol consumption status was assessed based on responses to the question, “Had at least 12 alcohol drinks in any one year?” Individuals who answered “yes” were classified as drinkers, while those who replied “no” were categorized as nondrinkers. Hypertension was defined by any one of the following criteria, including self-reported history of hypertension, utilization of antihypertensive medication, with an average systolic blood pressure (SBP) ≥140 mmHg and/or an average diastolic blood pressure (DBP) ≥90 mmHg (21). The diagnosis of COPD was based on a self-reported physician’s diagnosis (29), which was verified through a combination of three self-reported COPD questionnaire items: “Has a doctor ever told you that you have chronic bronchitis?”, “Has a doctor ever told you that you have emphysema?”, and “Has a doctor or other health professional ever told you that you have COPD?”. Participants who responded “yes” to any of these questions were diagnosed as having COPD, while those who answered “no” were diagnosed as without COPD. Except for the above-mentioned covariates, some essential laboratory test variables were also collected in the current study, including estimated glomerular filtration rate (eGFR), high-density lipoprotein cholesterol (HDL-C), low-density lipoprotein cholesterol (LDL-C), total cholesterol (TC), triglycerides (TG), serum creatinine (SCR), lactate dehydrogenase (LDH), fasting serum insulin (FINS), fasting plasma glucose (FPG), blood urea nitrogen (BUN), aspartate aminotransferase (AST), alanine aminotransferase (ALT), total bilirubin (TBil), γ-glutamyl transpeptidase (GGT), etc.

### Ascertainment of mortality outcomes

The NCHS utilized probabilistic matching techniques, employing various identifiers such as social security numbers and dates of birth, to link NHANES data with the NDI and obtain survival status information for participants. Follow-up for these participants was terminated on December 31, 2019. If no match was identified in the NDI, it was assumed that the individual was alive. This study analyzed both survival outcomes and survival duration. All-cause mortality was defined as death from any cause, with specific causes of death identified using the International Statistical Classification of Diseases, 10th Revision (ICD-10). Cardiovascular deaths were identified using the International Statistical Classification of Diseases, 10th Revision (ICD-10) codes (I00-I09, I11, I13, and I20-I51) (21, 22).

### Statistical analyses

Based on the NHANES analytic and reporting guidelines, complex sampling designs and sampling weights were taken into account during the analysis. Continuous variables were presented as median (Q1-Q3, interquartile) due to their non-normal distribution characteristics, while categorical variables were described as frequency and percentage. The Student *t* test or the Mann-Whitney U test, and the chi-square test or the Fisher’s exact test were utilized to identify the differences of continuous and categorical variables between two groups, respectively.

The optimal cut-off value for PNI, which got the most significant association with the survival outcomes, was identified by the methods of maximally selected rank statistics, which was completed by using the ‘maxstat’ package (30). Participants were divided into higher- and lower-PNI groups based on the identified PNI cut-off values. Weighted Cox regression analysis was performed to explore the association between PNI and all-cause and CVD mortality among in diabetic or prediabetic CVD patients. Two regression models were established for the adjustment of potential confounding factors, with the variables of age and gender being adjusted in model 1 and the variables of age, gender, race, education, marital status, FIR, family income-to-poverty ratio (FIR), smoking, drinking, BMI, COPD, hypertension, eGFR, HbA1C, TG and TC being adjusted in model 2. Subgroups analyses, interaction tests and restricted cubic spline (RCS) analyses, were conducted to verify the robustness and nonlinearity of the association between PNI and all-cause and CVD mortality in diabetic or prediabetic CVD patients. Survival probabilities were calculated by using the Kaplan-Meier method, and comparisons between groups were made by using the log-rank test (31). Time-dependent receiver operating characteristic (ROC) analysis (30, 32), completed by the ‘timeROC’ package, was performed to assess the predictive accuracy of the PNI at different time points for survival outcomes. All data were analyzed using R Software (version 4.4.1). A two-tailed p-value of less than 0.05 was considered to be statistically significant.

## Results

### Characteristics of the study population

A total of 1509 CVD participants with diabetes or prediabetes were included in the current study (**Figure 1**), and the median follow-up time for them was 61 (min to max, 1-162) months. The participants were categorized into the higher group (PNI ≥46.5, n=1211) and the lower group (PNI<46.5, n=298) based on the PNI cut-off value identified by maximally selected rank statistics (**Figure 2**). Compared with those in the lower PNI group, participants in the higher PNI group were more likely to be younger, be current drinkers, had no COPD, had higher levels of eGFR, TC, TG, albumin, serum iron and GGT. More details about the characteristics of diabetic or prediabetic CVD patients were presented in **Tables 1**, **2**.

**Table 1:** Characteristic of participants based on PNI grouping.

Variables	Total	Lower PNI (<46.5)	Higher PNI (≥46.5)	P-value
N	1509	298	1211	
Age	68.00 (60.00-76.00)	71.00 (63.00-79.75)	67.00 (60.00-75.00)	<0.001
Age categorical				<0.001
<60	332 (22.00%)	40 (13.42%)	292 (24.11%)	
≥60	1177 (78.00%)	258 (86.58%)	919 (75.89%)	
Gender				0.372
Male	860 (56.99%)	163 (54.70%)	697 (57.56%)	
Female	649 (43.01%)	135 (45.30%)	514 (42.44%)	
Race				0.156
Mexican American	186 (12.33%)	35 (11.74%)	151 (12.47%)	
Other Hispanic	130 (8.61%)	21 (7.05%)	109 (9.00%)	
Non-Hispanic White	717 (47.51%)	142 (47.65%)	575 (47.48%)	
Non-Hispanic Black	371 (24.59%)	86 (28.86%)	285 (23.53%)	
Other Race	105 (6.96%)	14 (4.70%)	91 (7.51%)	
Education				0.201
Less than high school	509 (33.73%)	113 (37.92%)	396 (32.70%)	
High school or equivalent	382 (25.31%)	74 (24.83%)	308 (25.43%)	
College or above	618 (40.95%)	111 (37.25%)	507 (41.87%)	
BMI	31.44 (27.50-36.00)	31.71 (27.63-35.76)	31.40 (27.50-36.02)	0.929
BMI categorical				0.491
<25	191 (12.66%)	40 (13.42%)	151 (12.47%)	
25-30	416 (27.57%)	74 (24.83%)	342 (28.24%)	
≥30	902 (59.77%)	184 (61.74%)	718 (59.29%)	
FIR	1.79 (1.03-2.82)	1.84 (1.11-2.45)	1.79 (1.02-2.96)	0.923
FIR categorical				0.008
<=1.0	355 (23.53%)	59 (19.80%)	296 (24.44%)	
1.0-3.0	801 (53.08%)	182 (61.07%)	619 (51.11%)	
≥3.0	353 (23.39%)	57 (19.13%)	296 (24.44%)	
Drinking				<0.001
No	841 (55.73%)	194 (65.10%)	647 (53.43%)	
Yes	668 (44.27%)	104 (34.90%)	564 (46.57%)	
Smoking				0.065
Never smoker	606 (40.16%)	108 (36.24%)	498 (41.12%)	
Former smoker	644 (42.68%)	145 (48.66%)	499 (41.21%)	
Current smoker	259 (17.16%)	45 (15.10%)	214 (17.67%)	
Hypertension				0.258
Yes	1221 (80.91%)	248 (83.22%)	973 (80.35%)	
No	288 (19.09%)	50 (16.78%)	238 (19.65%)	
COPD				0.002
No	1211 (80.25%)	220 (73.83%)	991 (81.83%)	
Yes	298 (19.75%)	78 (26.17%)	220 (18.17%)	
Diabetes	1213 (80.38%)	261 (87.58%)	952 (78.61%)	–
Prediabetes	296 (19.62%)	37 (12.42%)	259 (21.39%)	–

Date are presented as Median (Q1-Q3) or n (%). BMI, body mass index; FIR, family income-to-poverty ratio; COPD, chronic obstructive pulmonary disease.

**Table 2:** Baseline levels of laboratory characteristics based on PNI grouping.

Variable	Total	Lower PNI (<46.5)	Higher PNI (≥46.5)	P-value
N	1509	298	1211	
HbA1C, %	6.60 (5.90-7.60)	6.50 (6.00-7.70)	6.60 (5.90-7.60)	0.547
eGFR, mL/min/1.73m^2^	70.90 (52.13-88.78)	58.04 (37.11-76.78)	73.67 (55.82-90.96)	<0.001
LDL-C, mmol/L	2.33 (1.78-3.00)	2.22 (1.76-2.90)	2.38 (1.81-3.03)	0.349
HDL-C, mmol/L	1.14 (0.98-1.40)	1.16 (0.98-1.42)	1.14 (0.96-1.40)	0.306
TC, mmol/L	4.29 (3.62-5.09)	4.16 (3.39-4.93)	4.32 (3.70-5.13)	0.01
TG, mmol/L	1.67 (1.16-2.44)	1.49 (1.03-2.18)	1.74 (1.20-2.51)	<0.001
Scr, umol/L	90.17 (72.49-114.04)	105.64 (81.33-147.41)	88.40 (71.60-107.85)	<0.001
LDH, IU/L	140.00 (121.00-161.00)	145.07 (124.25-173.00)	139.00 (120.00-158.00)	<0.001
Uric acid, umol/L	356.90 (291.50-428.30)	374.70 (285.50-446.10)	356.90 (291.50-422.30)	0.004
Albumin, g/L	41.00 (38.00-43.00)	37.00 (35.00-39.00)	41.00 (40.00-43.00)	<0.001
FINS, pmol/L	131.39 (77.46-131.39)	131.39 (60.06-131.39)	131.39 (80.22-131.39)	0.084
FPG, mmol/L	8.18 (7.16-8.18)	8.18 (6.77-8.18)	8.18 (7.22-8.18)	0.667
Serum potassium, mmol/L	4.11 (3.90-4.40)	4.20 (3.90-4.50)	4.10 (3.90-4.40)	<0.001
Serum sodium, mmol/L	139.00 (138.00-141.00)	139.00 (137.00-141.00)	139.00 (138.00-141.00)	0.952
Serum iron, umol/L	13.10 (10.00-16.70)	11.40 (8.85-15.15)	13.40 (10.40-17.00)	<0.001
BUN, mmol/L	6.07 (4.64-8.21)	7.14 (5.00-10.71)	5.71 (4.28-7.85)	<0.001
AST (IU/L)	22.00 (18.00-27.00)	20.00 (17.00-26.00)	23.00 (19.00-28.00)	0.084
ALT(IU/L)	20.00 (15.00-27.00)	17.00 (14.00-22.00)	21.00 (16.00-28.00)	0.423
TBil, umol/L	10.26 (8.55-13.68)	10.26 (6.84-13.68)	10.26 (8.55-13.68)	0.8
GGT (IU/L)	24.00 (17.00-38.00)	22.50 (16.00-45.00)	24.00 (17.00-36.50)	0.041

Data are presented as Median (Q1-Q3). eGFR, estimated glomerular filtration rate; HDL-C, High-density lipoprotein cholesterol; LDL-C, Low-density lipoprotein cholesterol; TC, Total cholesterol; TG, Triglycerides; Scr, , serum creatinine; LDH, lactate dehydrogenase; FINS, Fasting serum insulin; FPG, fasting plasma glucose; BUN, Blood urea nitrogen; AST, Aspartate aminotransferase; ALT, Alanine Aminotransferase; TBil, total bilirubin; GGT, γ-glutamyl transpeptidase.

### Associations of the PNI with all−cause mortality

**Figure 3:**
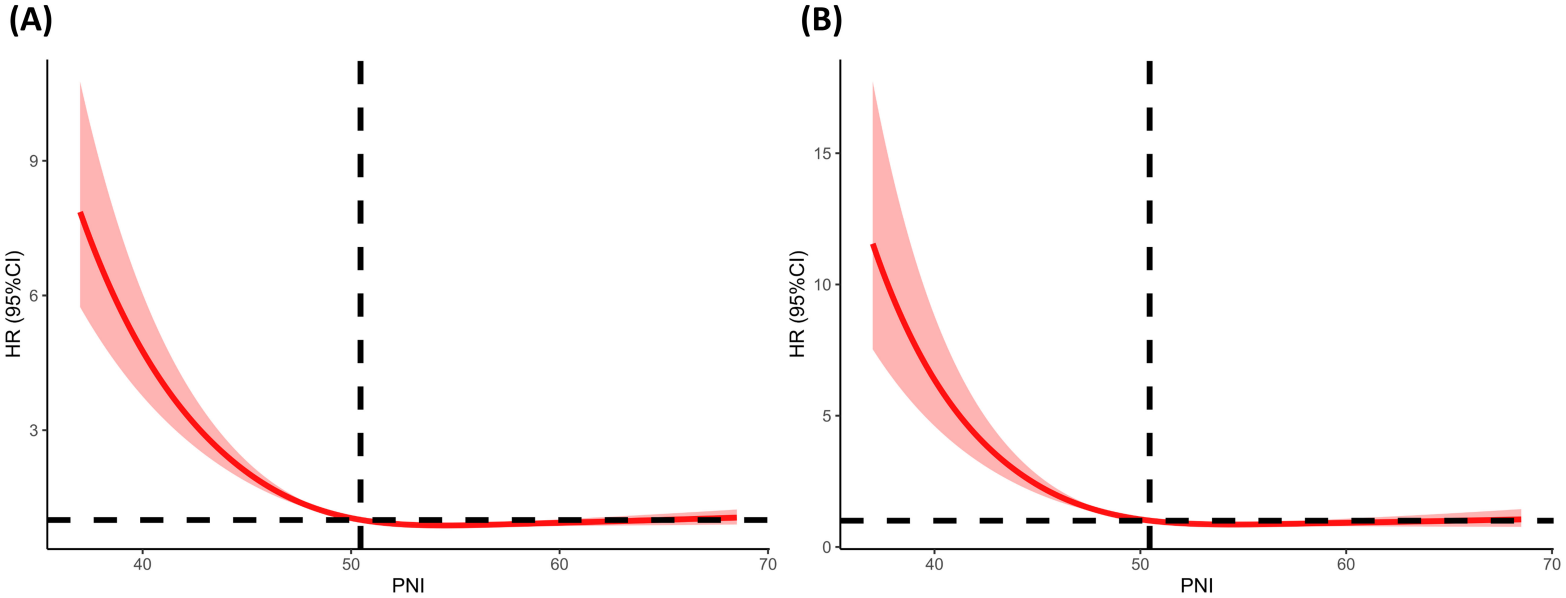
Determination of the association of PNI with all-cause and CVD mortality among diabetic or prediabetic CVD patients. **(A)** All-cause mortality; **(B)** CVD mortality. The RCS regression model was adjusted as model 2. The red solid line of the restricted cubic spline (RCS) curve represents the fitted smooth curve, while the shaded area indicates the 95% confidence interval. RCS, restricted cubic spline; PNI, prognostic nutritional index; CVD, cardiovascular disease; HR, hazard ratio.

During a median follow-up of 61 months (interquartile range (IQR), 33.0–103.0 months), a total of 507 (33.60%) diabetic or prediabetic CVD patients died, including 170 (33.53%) CVD deaths and 337 (66.47%) non-CVD deaths. Restricted cubic spline (RCS) analysis is commonly used in data analysis to explore the potential nonlinear relationships between an exposed variable and outcome variable. In this study, the results revealed a negative and nonlinear association between PNI and all-cause mortality in diabetic or prediabetic CVD patients (P for nonlinearity<0.05) (**Figure 3A**).

**Figure 4:**
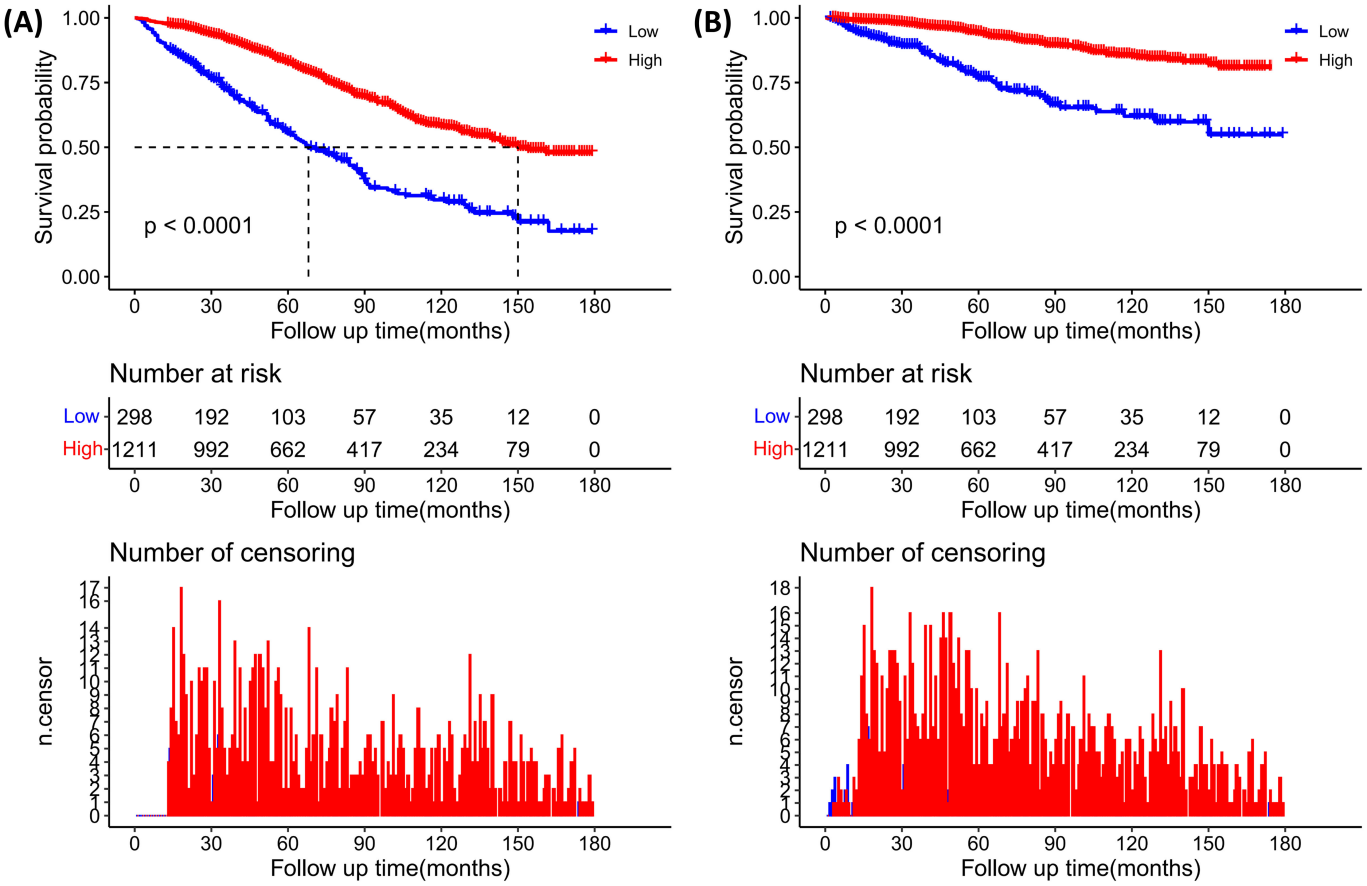
Kaplan–Meier curves of diabetic or prediabetic CVD patients within different PNI subgroups (Higher PNI, ≥46.5; Lower PNI, <46.5). **(A)** All-cause mortality; **(B)** CVD mortality. +9.

In the entire cohort, including diabetic or prediabetic CVD patients, weighted Cox regression analyses were further performed to explore the association between PNI and all-cause mortality. In the non-adjusted model (crude model), increased levels of PNI were observed to associate with reduced risks for all-cause mortality (PNI as continuous variable: HR=0.93, 95% CI: 0.91-0.95, P<0.0001; PNI as dichotomous variables: HR=0.38 for higher PNI group, 95% CI: 0.31-0.45, P<0.0001). After full adjustment of potential covariates (model 2), increased levels of PNI were also associated with decreased risks for all-cause mortality (PNI as continuous variable: HR=0.95, 95%CI: 0.94-0.97, P<0.0001; PNI as dichotomous variables: HR=0.49 for higher PNI group, 95%CI: 0.40-0.60, P<0.0001) (**Table 3**). According to the Kaplan-Meier survival analysis in all participants, those in the higher PNI group got significantly higher survival rates as opposed to those in the lower PNI group (**Figure 4A**).

**Table 3:** Association between PNI and mortality in diabetic or prediabetic CVD patients.

	Crude model	Model 1	Model 2
	HR (95% CI) P-value	HR (95% CI) P-value	HR (95% CI) P-value
all-cause mortality			
PNI	0.93 (0.91, 0.95) <0.0001	0.94 (0.92, 0.96) <0.0001	0.95 (0.94, 0.97) <0.0001
PNI categorical			
<46.5 (lower PNI)	1	1	1
≥46.5 (higher PNI)	0.38 (0.31, 0.45) <0.0001	0.41 (0.34, 0.50) <0.0001	0.49 (0.40, 0.60) <0.0001
CVD mortality			
PNI	0.90 (0.88, 0.93) <0.0001	0.91 (0.88, 0.94) <0.0001	0.93 (0.90, 0.96) <0.0001
PNI categorical			
<46.5 (lower PNI)	1	1	1
≥46.5 (higher PNI)	0.28 (0.21, 0.38) <0.0001	0.31 (0.23, 0.43) <0.0001	0.36 (0.26, 0.50) <0.0001

Crude Model, unadjusted;

Model 1, adjusted for age and gender;

Model 2, adjusted for age, gender, race, education, marital status, FIR, family income-to-poverty ratio (FIR), smoking, drinking, BMI, COPD, hypertension, eGFR, HbA1C, TG and TC.

In the diabetic CVD patients, the results from weighted Cox regression analyses revealed that increased levels of PNI were associated with reduced risks for all-cause mortality in all models. In the fully-adjusted model (model 2), higher PNI was significantly correlated with reduced risks for all-cause mortality (PNI as continuous variable: HR=0.94, 95%CI: 0.92-0.96, P<0.0001; PNI as dichotomous variables: HR=0.48 for higher PNI group, 95%CI: 0.39-0.59, P<0.0001) (**Supplementary Table 1**). Similar trends were observed in Kaplan-Meier survival curves, as presented in **Supplementary Figure 1A**.

In the prediabetic CVD patients, higher PNI was found to associate with reduced risk of all-cause mortality in the crude model (**Supplementary Table 2**, **Supplementary Figure 2A**) and model 1, but such association became insignificant in the fully-adjusted model (**Supplementary Table 2**).

### Associations of the PNI with CVD mortality

A negative and nonlinear association between PNI and CVD mortality was observed in diabetic or prediabetic CVD patients (P for nonlinearity<0.05) (**Figure 3B**).

In the entire cohort, weighted Cox regression analyses revealed that increased levels of PNI (as continuous and dichotomous variable) were significantly correlated with reduced risks for CVD mortality (**Table 3**). In the unadjusted model (Crude model), when analyzed as continuous variable, every 1-point increasement in PNI was associated with 10% reduced risk for CVD mortality (HR=0.90, 95%CI:0.88-0.93, P<0.0001). After adjusting for confounding factors, this association still remained significant (**Table 3**). Similarly, in the unadjusted model (Crude model), when analyzed as categorical variable, higher levels of PNI was significantly associated with decreased risks for CVD mortality (HR=0.36 for higher PNI group, 95%CI: 0.26-0.50, P<0.0001) (**Table 3**). The Kaplan-Meier survival plots revealed that the CVD mortality was lower in the higher PNI group than in the lower PNI group (**Figure 4B**).

In diabetic CVD patients, weighted multivariable Cox regression analyses indicated a significantly negative association between PNI and CVD mortality (**Supplementary Table 1**). In the unadjusted model (Crude model), when PNI was analyzed as continuous variable, every 1-point increase in PNI was correlated with 9% reduced risk of CVD mortality (HR=0.32, 95%CI: 0.23-0.44, P<0.0001). Similarly, in the fully-adjusted model, higher PNI was significantly associated with decreased risks for CVD mortality (HR=0.37 for higher PNI group, 95%CI: 0.26-0.52, P<0.0001) (**Supplementary Table 1**), which was confirmed by the findings from the Kaplan-Meier survival plots (**Supplementary Figure 1B**).

In the prediabetic CVD patients, although patients in the higher PNI group (PNI≥46.5) was associated with reduced risk for CVD mortality when PNI was analyzed as dichotomous variable in all Cox regression models. However, such association became insignificant when PNI was analyzed as continuous variable in the fully-adjusted model (**Supplementary Table 2**). Similarly, the Kaplan-Meier survival plots showed that the CVD mortality was lower in participants with a higher PNI level (PNI≥46.5) as opposed to participants with a lower PNI level (PNI<46.5) (**Supplementary Figure 2B**).

### Subgroup analyses

In order to confirm the robustness and stability of the association between PNI and all-cause and CVD mortality, subgroup analyses were conducted based on age, gender, smoking, drinking, COPD, BMI and hypertension, with the results being presented in **Tables 4**, **5**. A similar and consistent association was observed between PNI and all-cause and CVD mortality in different subgroups based on these variables. Additionally, no significant interactions between the aforementioned characteristics and PNI were found for all-cause and CVD mortality (all P interaction >0.05).

**Table 4:** Stratified analyses to verify the associations between PNI and all-cause mortality.

	All-cause mortality		P interaction
	HR (95% CI) P-value		
	Lower PNI (<46.5)	Higher PNI (≥46.5)	
Age			0.1260
<60	1	0.22 (0.10, 0.49) 0.0002	
≥60	1	0.52 (0.42, 0.65) <0.0001	
Gender			0.0814
Male	1	0.41 (0.32, 0.54) <0.0001	
Female	1	0.60 (0.42, 0.84) 0.0032	
Smoking			0.6780
Never	1	0.41 (0.29, 0.58) <0.0001	
Former	1	0.49 (0.37, 0.65) <0.0001	
Current	1	0.42 (0.22, 0.79) 0.0066	
Drinking			0.8428
No	1	0.48 (0.38, 0.62) <0.0001	
Yes	1	0.52 (0.36, 0.75) 0.0004	
COPD			0.9736
No	1	0.49 (0.39, 0.61) <0.0001	
Yes	1	0.46 (0.30, 0.70) 0.0004	
BMI			0.7123
<25	1	0.35 (0.20, 0.63) 0.0004	
25-30	1	0.48 (0.32, 0.72) 0.0004	
≥30	1	0.47 (0.36, 0.61) <0.0001	
Hypertension			0.9265
Yes	1	0.50 (0.40, 0.63) <0.0001	
No	1	0.49 (0.31, 0.78) 0.0027	

Variables of age, gender, race, education, marital status, FIR, family income-to-poverty ratio (FIR), smoking, drinking, BMI, COPD, hypertension, eGFR, HbA1C, TG and TC were adjusted in subgroup analysis and interaction test. BMI, body mass index, FIR, family income-to-poverty ratio; COPD, chronic obstructive pulmonary disease; DM, diabetes.

**Table 5:** Stratified analyses to verify the associations between PNI and CVD mortality.

	CVD mortality		P interaction
	HR (95% CI) P-value		
	Lower PNI (<46.5)	Higher PNI (≥46.5)	
Age			0.7034
<60	1	0.19 (0.04, 0.85) 0.0291	
≥60	1	0.37 (0.26, 0.52) <0.0001	
Gender			0.2231
Male	1	0.31 (0.20, 0.47) <0.0001	
Female	1	0.38 (0.21, 0.70) 0.0020	
Smoking			0.4791
Never	1	0.18 (0.10, 0.31) <0.0001	
Former	1	0.52 (0.30, 0.90) 0.0185	
Current	1	0.24 (0.08, 0.75) 0.0138	
Drinking			0.7344
No	1	0.38 (0.25, 0.57) <0.0001	
Yes	1	0.34 (0.17, 0.65) 0.0012	
COPD			0.2802
No	1	0.39 (0.27, 0.58) <0.0001	
Yes	1	0.22 (0.10, 0.46) <0.0001	
BMI			0.7636
<25	1	0.18 (0.07, 0.46) 0.0004	
25-30	1	0.32 (0.15, 0.66) 0.0020	
≥30	1	0.43 (0.27, 0.66) 0.0002	
Hypertension			0.885
Yes	1	0.37 (0.25, 0.55) <0.0001	
No	1	0.30 (0.14, 0.63) 0.0015	

Variables of age, gender, race, education, marital status, FIR, family income-to-poverty ratio (FIR), smoking, drinking, BMI, COPD, hypertension, eGFR, HbA1C, TG and TC were adjusted in subgroup analysis and interaction test. BMI, body mass index, FIR, family income-to-poverty ratio; COPD, chronic obstructive pulmonary disease; DM, diabetes.

### Identification of the predictive value of PNI for all-cause and CVD mortality based on Time-dependent ROC analysis

**Figure 5:**
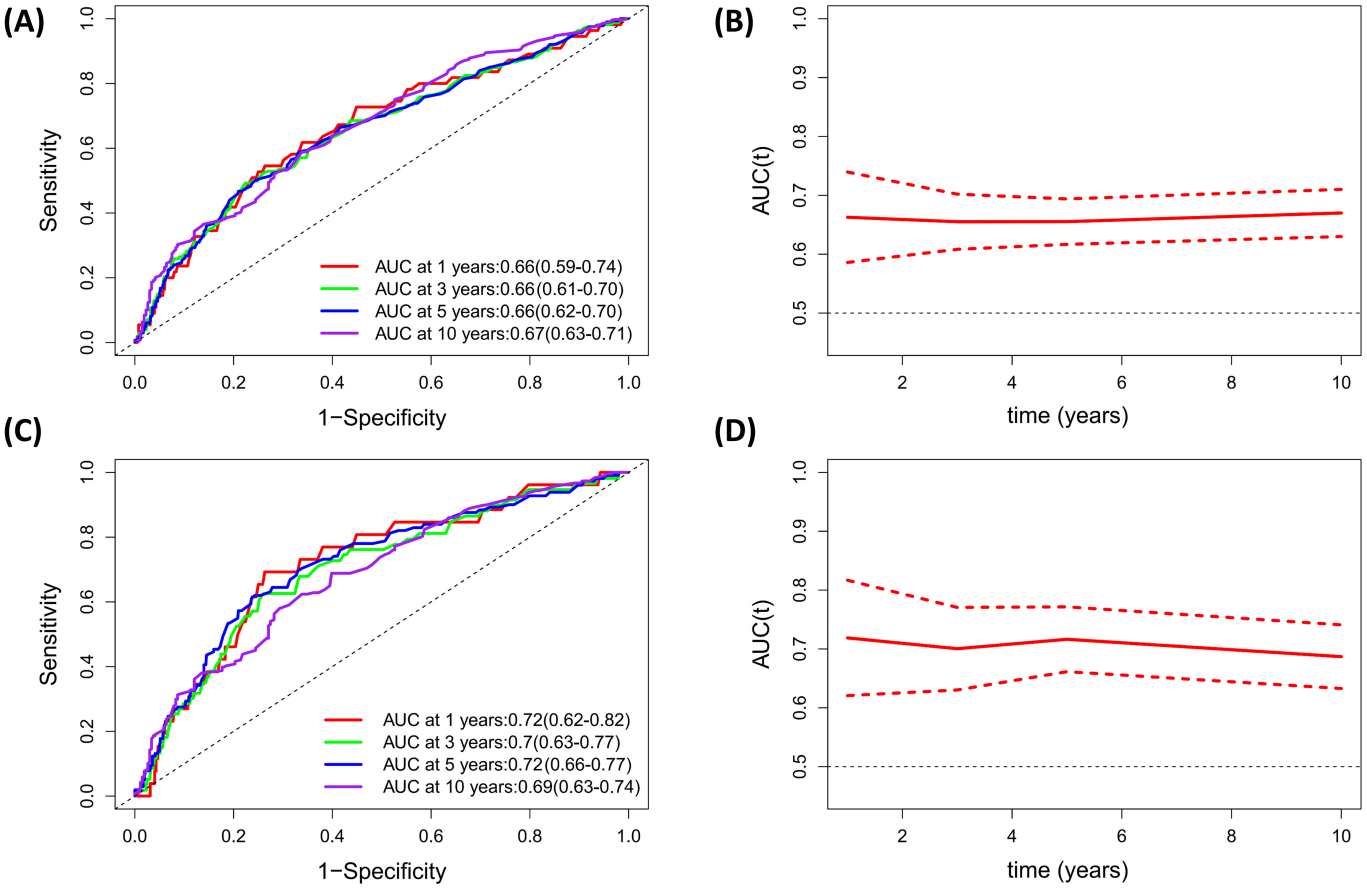
Evaluation of predicting performance of PNI for all-cause mortality and CVD mortality based on Time-dependent ROC curves and time-dependent AUC curves. **(A, B)** All-cause mortality; **(C, D)** CVD mortality.

The time-dependent ROC analyses were conducted to assess the prognostic value of PNI for all-cause and CVD mortality in diabetic or prediabetic CVD patients. The results indicated that the area under the curve (AUC) of PNI for 1-year, 3-year, 5-year and 10-year all-cause mortality was 0.66 (0.59-0.74), 0.66 (0.61-0.70), 0.66 (0.62-0.70), and 0.67 (0.63-0.71), respectively (**Figures 5A, B**). Regarding the CVD mortality, the AUC of PNI for 1-year, 3-year, 5-year and 10-year was 0.72 (0.62-0.82), 0.70 (0.63-0.77), 0.72 (0.66-0.77), and 0.69 (0.63-0.74), respectively (**Figures 5C, D**). These findings suggest that the PNI holds significant predictive potential for both all-cause and CVD mortality over short and long durations.

## Discussion

In the current study, we aimed to elucidate the association of PNI, an immunonutritional biomarker, with all-cause and CVD mortality in CVD patients with diabetes or prediabetes. Our results revealed that higher level of PNI was significantly associated reduced risk for all-cause and CVD mortality, and the relationship between PNI and mortality was non-linear (L-shape). The association remained stable across different subgroups based on variables of age, gender, smoking, drinking, COPD, BMI and hypertension, and was not modified by the aforementioned characteristics. As survival predictor, the time-dependent ROC curve revealed that PNI performed well in predicting the 1-, 3-, 5-, and 10-year survival rates of all-cause and CVD mortality.

Cardiovascular diseases represent the primary cause of mortality worldwide, with their prevalence steadily increasing in developed and developing countries (33). Individuals with insulin resistance and diabetes face an even greater susceptibility to cardiovascular diseases (19). Both CVD and diabetes/prediabetes were closely related to nutritional imbalance and immune disorders, and the two aspects were mutually linked to each other. Detailedly speaking, when the body encounters an abundance of nutrients, it reserves them as energy in adipose tissue, the liver, muscle, and other unwanted locations (e.g., cardio-cerebrovascular system, the primary cause of cardiovascular and cerebrovascular diseases). Conversely, during episodes of nutrient scarcity, the body mobilizes these stored reserves to sustain physiological functions. This process leads to variations in adipose tissue volume, which can either augment or diminish depending on nutrient availability. Such fluctuations influence the secretion of various adipose tissue hormones and adipocytokines, including leptin, tumor necrosis factor-alpha (TNF-α), interleukin-6 (IL-6), and adiponectin. A number of these adipocytokines serve crucial roles in immune signaling, which can modify the biological characteristics of immune cells and fine-tune the immune response. Therefore, immunonutritional indicators which can accurately represent the dynamic changes of immune and nutritional status of individuals can serve as effective prognosticator for predicting all-cause and CVD mortality in diabetic and prediabetic CVD patients.

In our study, we found that PNI was non-linearly associated with mortality in diabetic and prediabetic CVD patients, and a threshold of 46.5 for PNI was identified by maximally selected rank statistics analysis. Individuals with higher PNI level (>46.5) showed significantly lower mortality risk, indicating that enhancing immunonutritional status can help to improve prognosis in CVD patients with diabetes or prediabetes. A previous meta-analysis involving seven studies found that, among patients with acute coronary syndrome, those with lower PNI levels exhibited a pooled hazard ratio (HR) of 2.97 (95%CI: 1.65–5.34, P=0.0003, I²=89%) for all-cause mortality and 2.04 (95%CI: 1.59–2.61; P<0.00001; I²=21%) for major adverse cardiovascular events (34). The cut-off value of PNI in the aforementioned studies ranged from 45 to 56, which was very similar to the threshold (PNI=46.5) identified in our study. Furthermore, numerous studies have investigated the relationship between PNI tertiles or quartiles and mortality, consistently concluding that higher PNI levels are associated with a reduced risk of mortality (35). These results collectively underscored the significant predictive potential of PNI as a prognostic indicator for patients with cardiovascular disease.

PNI is derived from albumin (ALB) and lymphocyte, among which ALB is a bioactive substance produced by the liver and plays vital roles in various aspects, while lymphocyte is an important component of the immune system. ALB is reported to implicate in various conditions, including diabetes (36), sepsis (37), ischemic stroke (38, 39), Alzheimer’s disease (40, 41), etc. In a 2465-person cohort study, low ALB levels and increased nutritional risk is independently associated with the 30-day mortality in patients with acute disease, with the AUC being 0.77 and 0.75, respectively (42). Besides, both malnutrition and albumin levels emerged as independent prognostic factors for end-stage kidney disease mortality during a 10-year follow-up (43). As an pivotal component of PNI, lymphocytes are closely involved in the onset and progression of multiple diseases, including diabetes and CVD (44, 45). A fundamental study has uncovered that the intake of excessive nutrients can promote the production of abundant inflammatory cytokines in immune cells (e.g., macrophages), which is the primary cause for continuous chronic inflammation and insulin resistance, the initiator and accelerator of diabetic or prediabetic status (46).

In our study, the interaction between PNI and other covariates are not significant, suggesting that PNI is an independent predictor of mortality. Actually, except for the population mentioned in our study, PNI was reported to serve as valuable predictor for the outcome (mainly the risks for mortality, increased risks for the occurrence of comorbidities) in other participants populations, such as chronic kidney disease (CKD) (47), type 2 diabetes (17), pheochromocytoma (48), osteoporosis (49), Crimean Congo hemorrhagic fever (50), and so on. The findings from our study, as well as from previous studies, highlighted the fundamental roles of immunity and nutrition in multiple disease conditions. It should be noted that PNI may be not a highly-specific indicator for certain disease, but its’ favorable prediction performance and remarkable association with various diseases have emphasized its’ useful predictive information and intervention target in clinical practice. Therefore, improving the immunonutritional status may help to get better prognosis when we are treating the targeted disease.

Our study did not found significant association between PNI and mortality in prediabetic CVD patients, which may be associated with the variations in serum albumin. A prior study examining the immune and inflammatory response profiles in individuals with prediabetes and diabetes found that lymphocyte levels increased in association with subclinical disease. However, these levels remained relatively stable despite the progression to diabetes (51). Thus, while lymphocyte counts may rise with earlier immune disturbances, they do not significantly fluctuate with the advancement of the disease. This suggested that the changes of albumin may contribute to the prognostic difference of PNI in prediabetic and diabetic CVD patients. Actually, patients with prediabetes generally exhibit significantly lower levels of kidney function impairment compared to those with diabetes. This difference arises primarily because individuals with diabetes may experience proteinuria due to diabetic nephropathy, which leads to considerable loss of serum albumin and ultimately results in hypoalbuminemia. In our study, we specifically observed a significant difference in serum albumin levels between diabetic CVD patients and their prediabetic counterparts, with values of 40.26 ± 3.60 for diabetic patients versus 41.21 ± 3.61 for prediabetic patients (P < 0.001). Furthermore, it is important to note that many indicators used to evaluate the clinical prognosis of diabetes have incorporated serum albumin in their assessments, such as red blood cell distribution width (RDW)-to-albumin ratio (RAR) (52), neutrophil percentage-to-albumin ratio (NPAR) (53), and so on. This further highlights its vital predictive value in the outcomes related to diabetes. Considering all these factors, variations in albumin levels between diabetic and prediabetic patients may explain the predictive differences in PNI between the two groups.

A principal strength of our study lies in its incorporation of a substantial and diverse sample size, which not only bolsters the robustness of our conclusions but also significantly enhances statistical power. Moreover, as all participants were sourced from the NHANES survey, this design significantly minimizes the potential for selection bias. Nonetheless, several limitations to this study should be acknowledged. First, while we considered a range of potential confounding factors in our analysis, the possibility persists that PNI may be influenced by additional, unidentified variables, such as the eating patterns or medication usage. Second, given that this study primarily focused on individuals with diabetes or prediabetes in the United States, further research is essential to evaluate the extent to which our findings can be extrapolated to other populations. Third, as our study relies solely on baseline PNI data obtained from the NHANES database, it focuses exclusively on the prognostic value of baseline PNI. Thus, it remains unclear whether changes in PNI over time during follow-up can also serve as predictors of mortality, and this aspect necessitates further investigation. Fourth, the use of self-reported data to diagnose diabetes or cardiovascular disease (CVD) may introduce some misclassification bias in the current study. Fifth, since PNI is calculated based on serum albumin and lymphocyte counts, it cannot be considered a specific biomarker for prognostic prediction. Additionally, PNI exhibited an AUC ranging from 0.66 to 0.72, indicating moderate discrimination accuracy in assessing CVD or all-cause mortality among individuals with CVD and diabetes. Therefore, its use alone may not be adequate, and it is advisable to combine PNI with other prognostic indicators for clinical application. Finally, the median follow-up duration of 61 months is relatively short, which may be inadequate to capture mortality outcomes in all subjects and could potentially influence the findings of our study.

In summary, our study revealed a significant correlation between low PNI levels and heightened mortality in cardiovascular disease (CVD) patients with diabetes. These findings underscore the imperative to tackle malnutrition within the diabetic population, particularly among those afflicted by CVD. Preventive strategies should focus on obesity alleviation, whereas for individuals already experiencing malnutrition, efforts must prioritize the enhancement of nutritional status to reduce CVD-related mortality.

## Conclusion

Our study determined a significant and non-linear relationship between PNI and all-cause and CVD mortality in diabetic and prediabetic CVD patients. These findings suggest that PNI can be integrated into routine clinical practice as a reference tool for healthcare professionals when assessing the mortality risk in CVD patients with diabetes or prediabetes.

## Data Availability

The original contributions presented in the study are included in the article/**Supplementary Material**. Further inquiries can be directed to the corresponding author.
