# Supplementary material for: Associations of prognostic nutritional index with cardiovascular all-cause mortality among CVD patients with diabetes or prediabetes: evidence from the NHANES 2005–2018
Source: Front Immunol. 2025 Feb 12;16:1518295. doi: 10.3389/fimmu.2025.1518295 (PMC11860081; doi:10.3389/fimmu.2025.1518295)
Supplement: Supplementary file 1 [file Table1.docx]

**Supplementary Table 1. Association between PNI and mortality in diabetic CVD patients.**

|  | **Crude model** | **Model 1** | **Model 2** |
| --- | --- | --- | --- |
|  | **HR (95% CI) P-value** | **HR (95% CI) P-value** | **HR (95% CI) P-value** |
| **All-cause mortality** |  |  |  |
| PNI | 0.92 (0.90, 0.94) <0.0001 | 0.92 (0.91, 0.94) <0.0001 | 0.94 (0.92, 0.96) <0.0001 |
| PNI categorical |  |  |  |
| <46.5 (lower PNI) | 1 | 1 | 1 |
| ≥46.5 (higher PNI) | 0.39 (0.32, 0.48) <0.0001 | 0.41 (0.34, 0.51) <0.0001 | 0.48 (0.39, 0.59) <0.0001 |
| **CVD-mortality** |  |  |  |
| PNI | 0.91 (0.88, 0.94) <0.0001 | 0.91 (0.88, 0.94) <0.0001 | 0.92 (0.89, 0.95) <0.0001 |
| PNI categorical |  |  |  |
| <46.5 (lower PNI) | 1 | 1 | 1 |
| ≥46.5 (higher PNI) | 0.32 (0.23, 0.44) <0.0001 | 0.33 (0.24, 0.46) <0.0001 | 0.37 (0.26, 0.52) <0.0001 |

**Note:** Crude Model, unadjusted;

Model 1, adjusted for age and gender;

Model 2, adjusted for age, gender, race, education, marital status, FIR, family income-to-poverty ratio (FIR), smoking, drinking, BMI, COPD, hypertension, eGFR, HbA1C, TG and TC.

**Supplementary Table 2. Association between PNI and mortality in prediabetic CVD patients.**

|  | **Crude model** | **Model 1** | **Model 2** |
| --- | --- | --- | --- |
|  | **HR (95% CI) P-value** | **HR (95% CI) P-value** | **HR (95% CI) P-value** |
| **All-cause mortality** |  |  |  |
| PNI | 1.00 (0.97, 1.03) 0.8789 | 1.01 (0.99, 1.02) 0.4067 | 1.00 (0.99, 1.02) 0.5851 |
| PNI categorical |  |  |  |
| <46.5 (lower PNI) | 1 | 1 | 1 |
| ≥46.5 (higher PNI) | 0.33 (0.19, 0.58) 0.0001 | 0.48 (0.27, 0.85) 0.0123 | 0.61 (0.32, 1.14) 0.1191 |
| **CVD-mortality** |  |  |  |
| PNI | 0.88 (0.77, 1.01) 0.0600 | 0.92 (0.80, 1.07) 0.2904 | 0.99 (0.86, 1.14) 0.8867 |
| PNI categorical |  |  |  |
| <46.5 (lower PNI) | 1 | 1 | 1 |
| ≥46.5 (higher PNI) | 0.12 (0.03, 0.42) 0.0008 | 0.20 (0.06, 0.72) 0.0137 | 0.02 (0.00, 0.06) <0.0001 |

**Note:** Crude Model, unadjusted;

Model 1, adjusted for age and gender;

Model 2, adjusted for age, gender, race, education, marital status, FIR, family income-to-poverty ratio (FIR), smoking, drinking, BMI, COPD, hypertension, eGFR, HbA1C, TG and TC.


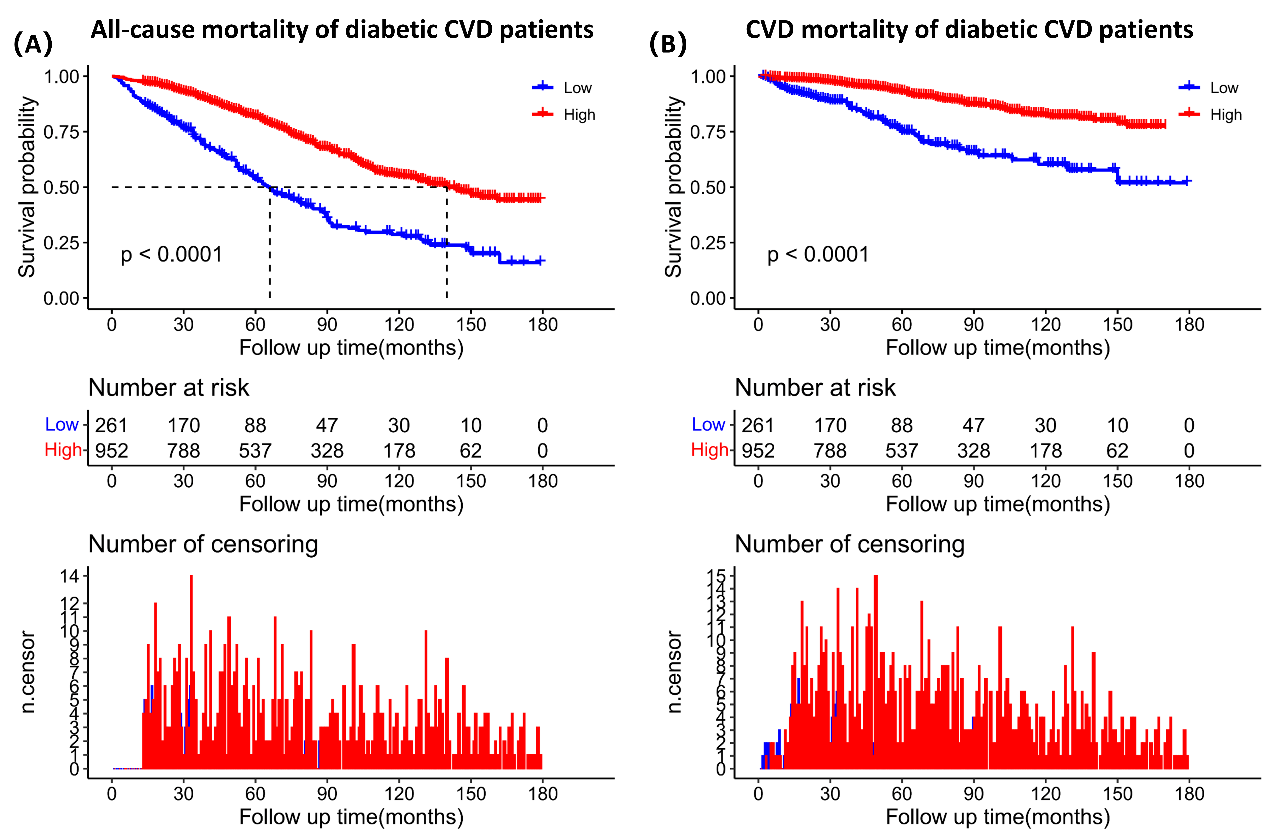


**SFigure 1.** Kaplan-Meier curves of all-cause and CVD mortality for the two subgroups PNI in diabetic CVD patients. (A) All-cause mortality of diabetic CVD patients; (B) CVD mortality of diabetic CVD patients.


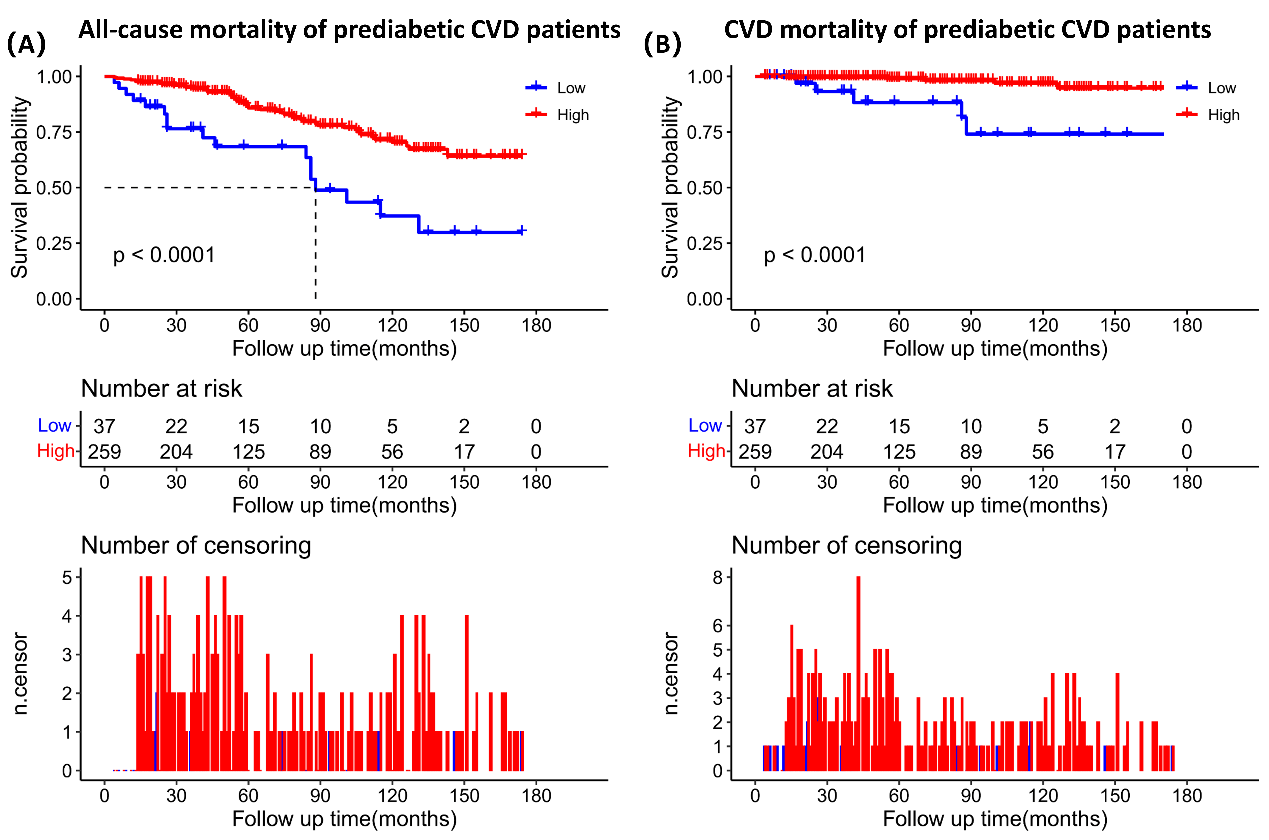


**SFigure 2.** Kaplan-Meier curves of all-cause and CVD mortality for the two subgroups PNI in prediabetic CVD patients. (A) All-cause mortality of prediabetic CVD patients; (B) CVD mortality of prediabetic CVD patients.
